# Supplementary material for: GWAS reveals determinants of mobilization rate and dynamics of an active endogenous retrovirus of cattle
Source: Nat Commun. 2024 Mar 9;15:2154. doi: 10.1038/s41467-024-46434-1 (PMC10924933; doi:10.1038/s41467-024-46434-1)
Supplement: Supplementary file 1 — Supplementary information [file 41467_2024_46434_MOESM1_ESM.pdf]

Supplementary Information for

GWAS reveals determinants of mobilization rate and dynamics of an active endogenous retrovirus of cattle

Lijing Tang[1*], Benjamin Swedlund[1,2], Sébastien Dupont[1], Chad Harland[1,3], Gabriel Costa Monteiro Moreira[1], Keith Durkin[1,4], Maria Artesi[1,4], Eric Mullaart[5], Arnaud Sartelet[1,6], Latifa Karim[1,7], Wouter Coppieters[1,7], Michel Georges[1*], Carole Charlier[1*]

[1]Unit of Animal Genomics, GIGA & Faculty of Veterinary Medicine, University of Liège, Belgium;
[2]Keck School of Medicine, University of Southern California, USA;
[3]Livestock Improvement Corporation, Hamilton, New Zealand;
[4]Laboratory of Human Genetics, GIGA & Faculty of Medicine, University of Liège, Belgium;
[5]CRV, Arnhem, The Netherlands;
[6]Comparative Veterinary Medicine, FARAH & Faculty of Veterinary Medicine, University of Liège, Belgium;
[7]Genomics core facility, GIGA, University of Liège, Belgium.

Correspondence:

Carole Charlier (carole.charlier@uliege.be)
Michel Georges (michel.georges@uliege.be)
Lijing Tang (lijing.tang@uliege.be)

Supplementary Information Content

Supplementary Figure 1:
Identification and functional validation of the causative mutation for CD.

Supplementary Figure 2:
Genomic features of polymorphic ERV elements.

Supplementary Figure 3:
Pedigree-based identification of five *de novo* insertions of ERVK[2-1-LTR] elements.

Supplementary Figure 4:
Assessing the repeatability and robustness of PCIP and lack of evidence of an effect of a bull's inbreeding coefficient on the rate of ERVK[2-1-LTR] mobilization in its germline.

Supplementary Figure 5:
Genomic landscape of ERVK[2-1-LTR] *de novo* insertions.

Supplementary Figure 6:
Genomic features of ERVK[2-1-LTR] *de novo* insertions.

Supplementary Figure 7:
Additional GWAS loci and genotypic effect for the eight significant loci.

Supplementary Figure 8:
Dot plot between sequences representing the C clade (X-axis) and D clade (Y-axis) and neighbor-joining tree obtained with the concatenated *GAG*-shared (55 base pairs) and *ENV*-shared (195 base pairs) segments.

Supplementary Figure 9:
Protein sequences and domain annotation of a representative element of the C-clade (chr19: 50,466,809 bp).

Supplementary Figure 10:
Examples of APOBEC3 mutational signature, recombination events and attrition at the boundaries.

Supplementary Figure 11:
Distribution of allelic frequencies of ERVK[2-1-LTR] segregating in Belgian Blue cattle.

Supplementary Figure 12:
Phylogenetic relationship with exogenous retroviruses and functional sequence features of ERVK[2-1-LTR].

Supplementary Figure 13:
Epistatic interaction between ERVK[2-1-LTR] elements with coding variants in the *GAG*, *PRO*, *POL* or *ENV* gene, and ERVK[2-1-LTR] elements without.

Supplementary Figure 14:
Comparison between the real and simulated frequency distribution of the resampling rate of ERVK[2-1-LTR] *de novo* mobilization events in BB bulls BE157971524, BE187351114, BE63811423.

Supplementary Figure 15:
Unselected examples of the evolution of the number of ERV elements (per genome) in a panmictic population of 1,000 animals over a course of 5,000 generations.

Supplementary Figure 16:
Schematic representation of the features exploited by *LocaTER* to identify polymorphic ERV element absent from the bovine reference sequence.

Supplementary Table 1:
Detailed domain annotation of a representative element of the C-clade (chr19: 50,466,809 bp).

Supplemental Method 1:
LocaTER for the detection of polymorphic and *de novo* repeat insertions.

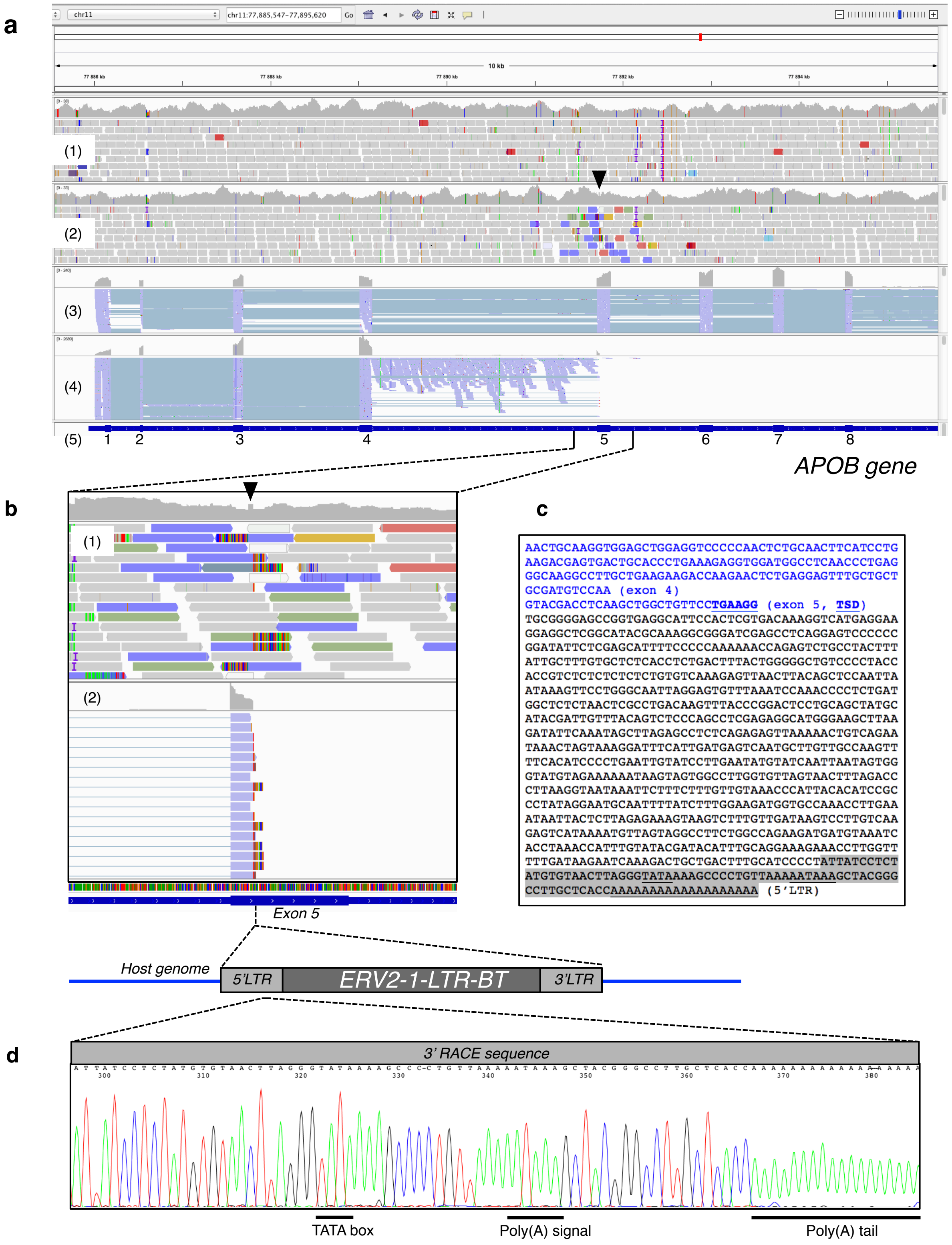

Supplementary Figure 1: Identification and functional validation of the causative mutation for CD.

(a) Integrative Genome Viewer (IGV) screen capture of a 10 kb genomic region (chr11:77,885,547-77,885,620bp) encompassing the ERVK insertion (black triangle) in the coding exon 5 of the *APOB* gene; with from top to bottom: the whole genome sequence (WGS) BAM files of a homozygous wild-type (1) and a heterozygous carrier (2) with reads from discordant read pairs surrounding the ERVK insertion showing a multicolor display;

liver cDNA sequence reads (light blue) for a homozygous wild-type (3) and a homozygous mutant (4) highlighting the transcriptional shut-off in *APOB* exon 5; genomic organization of the *APOB* gene with exons and introns displayed as thick and thin dark blue rectangles respectively (5). **(b)** IGV screen capture of a zoomed 500 bp region surrounding the ERVK insertion site; with - from top to bottom: the WGS of a heterozygous carrier (1) with target site duplication (TSD) pinpointed by a black triangle; reads from the allele without the insertion are shown in grey; reads from discordant read pairs surrounding the breakpoints are showing a multicolor display and reads overlapping the breakpoints a characteristic soft-clipped feature (lack of homology with the reference sequence); cDNA sequence reads (light blue) for a homozygous mutant (2) highlighting the transcriptional shut-off in *APOB* exon 5 and reads overlapping the breakpoint showing a characteristic soft-clipped feature. **(c)** Liver cDNA sequence from a 3'RACE experiment performed on a homozygous mutant; with coding exon 4 and 5 in dark blue; the TSD is shown in blue, bold and underlined; from the ERVK insertion site, the transcribed part of its 5' LTR sequence is presented in black, ending with a poly(A) tail preceded by canonical TATA box and 'AATAAA' poly(A) signal (underlined); the sequence boxed in dark grey corresponds to the Sanger cDNA sequence trace displayed in panel d**.** **(d)** Sanger cDNA sequence trace from the above 3'RACE experiment, highlighting TATA box, poly(A) signal and poly(A) tail with black horizontal bars; a schematic representation of the full-length ERVK insertion in *APOB* exon 5 is depicted above the sequence trace.

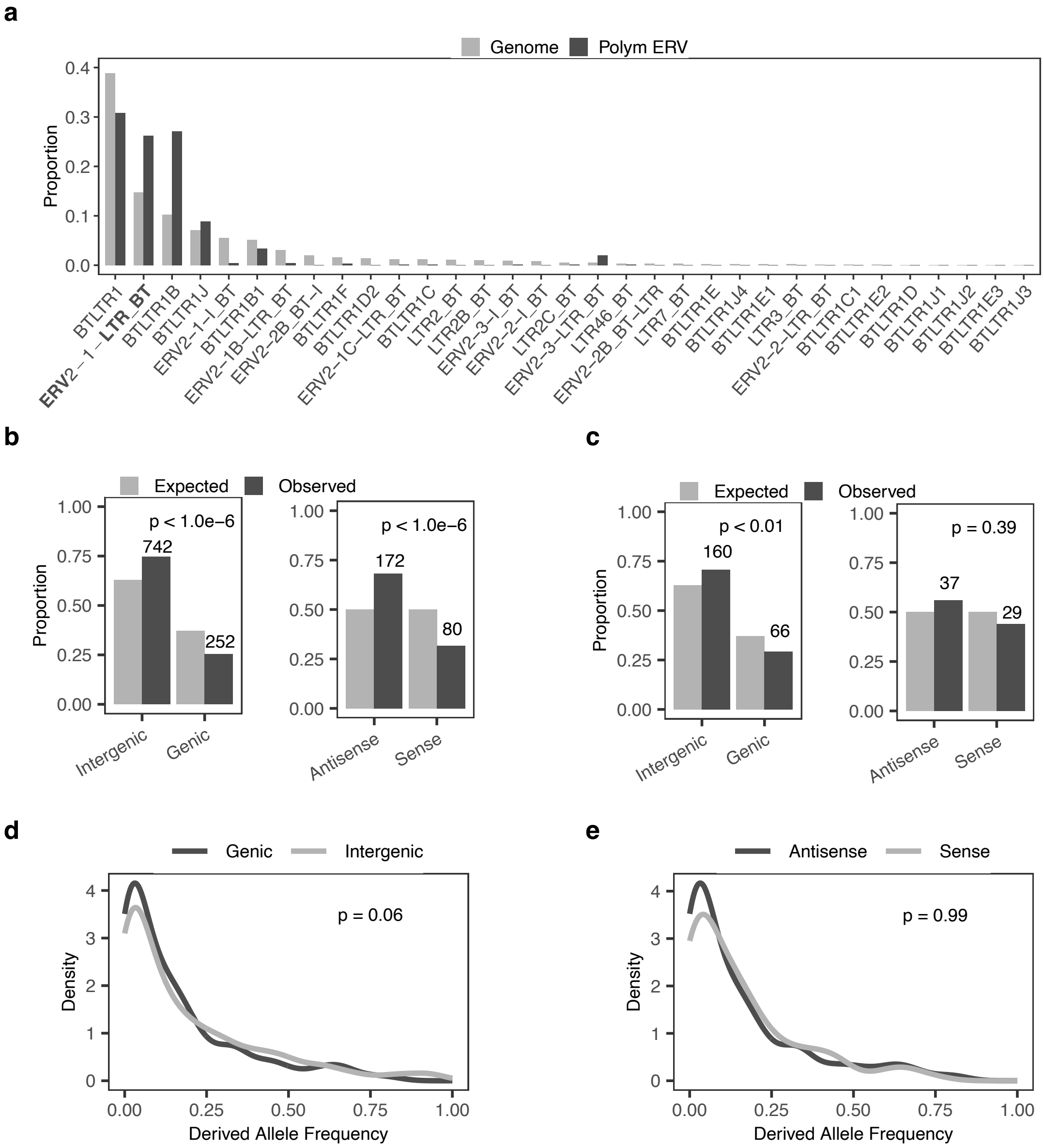

Supplementary Figure 2: Genomic features of polymorphic ERV elements.
(a) Proportional representation of ERVK sub-groups in genome space (light grey bars) and amongst polymorphic elements detected by *LocaTER* (dark grey bars). Repbase reports 33 subgroups of ERVK, of which the most abundant in the reference genome are BTLTR1 (38.9%), ERVK[2-1-LTR] (20.2%), BLTR1B (10.2%) and BLTR1J (7.1%). While BTLTR1 is underrepresented amongst polymorphic ERVK elements (30.1%), ERVK[2-1-LTR] (26.6%), BLTR1B (27.1%) and BLTR1J (8.8%) are respectively overrepresented 1.3, 2.6 and 1.2 times. Of note, the very rare ERVK[2-3-LTR] subgroup (0.5%) is 4 times overrepresented amongst polymorphic ERVK elements. This suggests that the latter four subgroups, especially, might still be active. **(b)** Genomic distribution of polymorphic ERVK elements (dark grey) compared to the corresponding genome space (light grey). Left: intergenic versus genic space. Right: antisense versus sense orientation for genic elements.
(c) Genomic distribution of polymorphic ERV elements other than the ERVK group (dark grey) compared to the corresponding genome space (light grey). Left: intergenic versus genic space. Right: antisense versus sense orientation for genic elements. The respective proportions did not differ significantly between ERVK and non-ERVK elements. See also main text. **(d)** Derived Allele Frequency (DAF) spectrum for genic (dark grey) and intergenic

(light grey) ERV elements. DAF of genic insertions are slightly ($p = 0.06$) shifted towards lower values as expected under purifying selection. **(e)** Derived Allele Frequency (DAF) spectrum for genic antisense (dark grey) and genic sense (light grey) ERV elements. There is no significant evidence (Wilcoxon sum of rank test) $p = 0.99$) for a shift of DAF of sense insertions towards lower values when compared to antisense, as expected if they would be subject to stronger purifying selection. Source data are provided as a Source Data file.

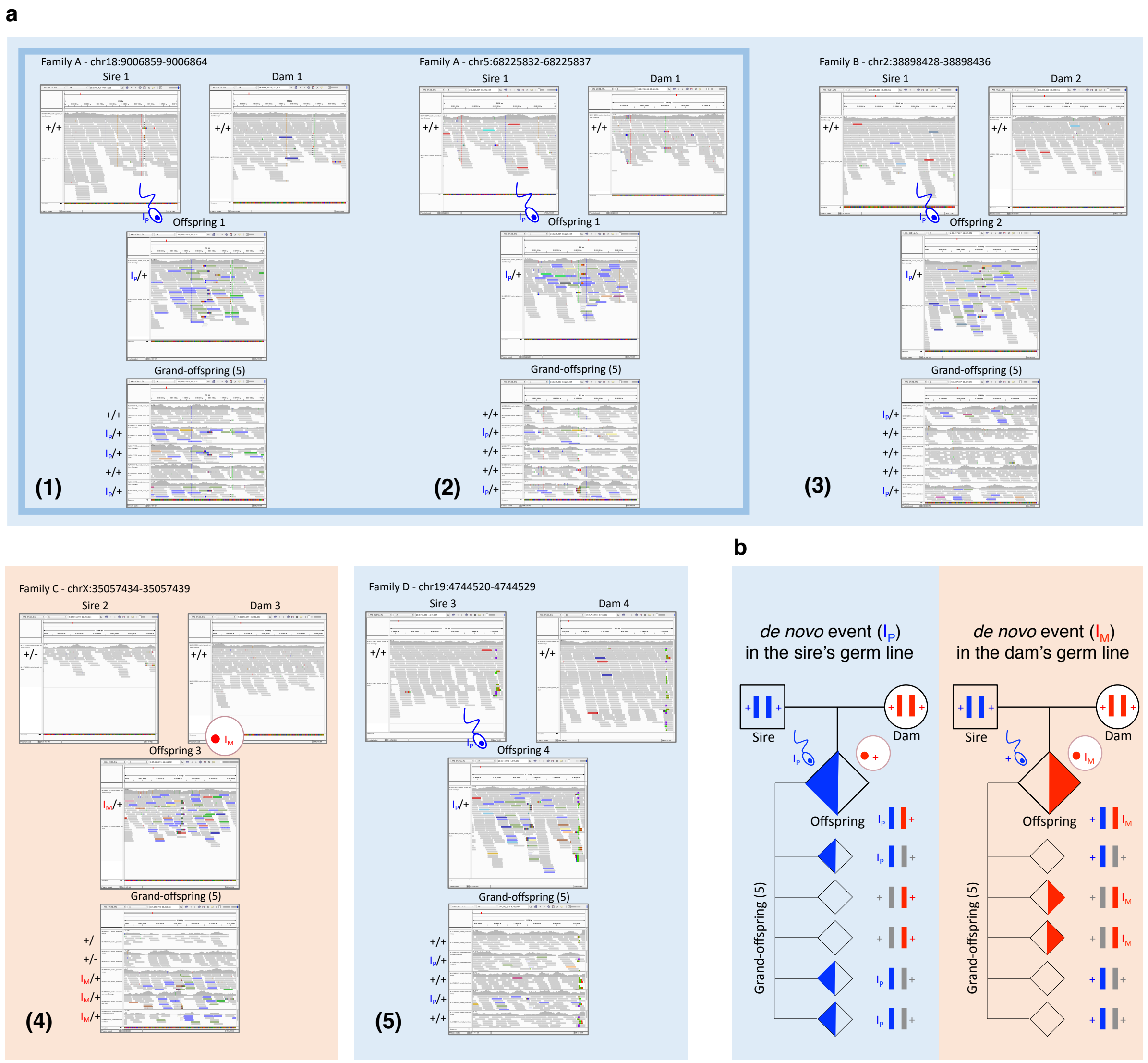

Supplementary Figure 3: Pedigree-based identification of five *de novo* insertions of ERVK[2-1-LTR] elements.

(a) IGV screen captures of the WGS corresponding to the respective genomic regions surrounding each of the five de novo insertions (1 to 5); with - from top to bottom - absence of the ERVK[2-1-LTR] element in the parents, presence in the offspring and transmission to grand-offspring in perfect linkage disequilibrium; a dark blue border highlights the family with two insertions transmitted by the same sperm cell; blue and pink backgrounds feature *de novo* events occurring in the paternal or maternal germline respectively; '+' for wild-type allele and 'I' for allele with insertion. Sires and Dams are numbered as in main Fig. 2d. **(b)** Schematic drawing of *de novo* events occurring either in the paternal (left) or maternal (right) germline.

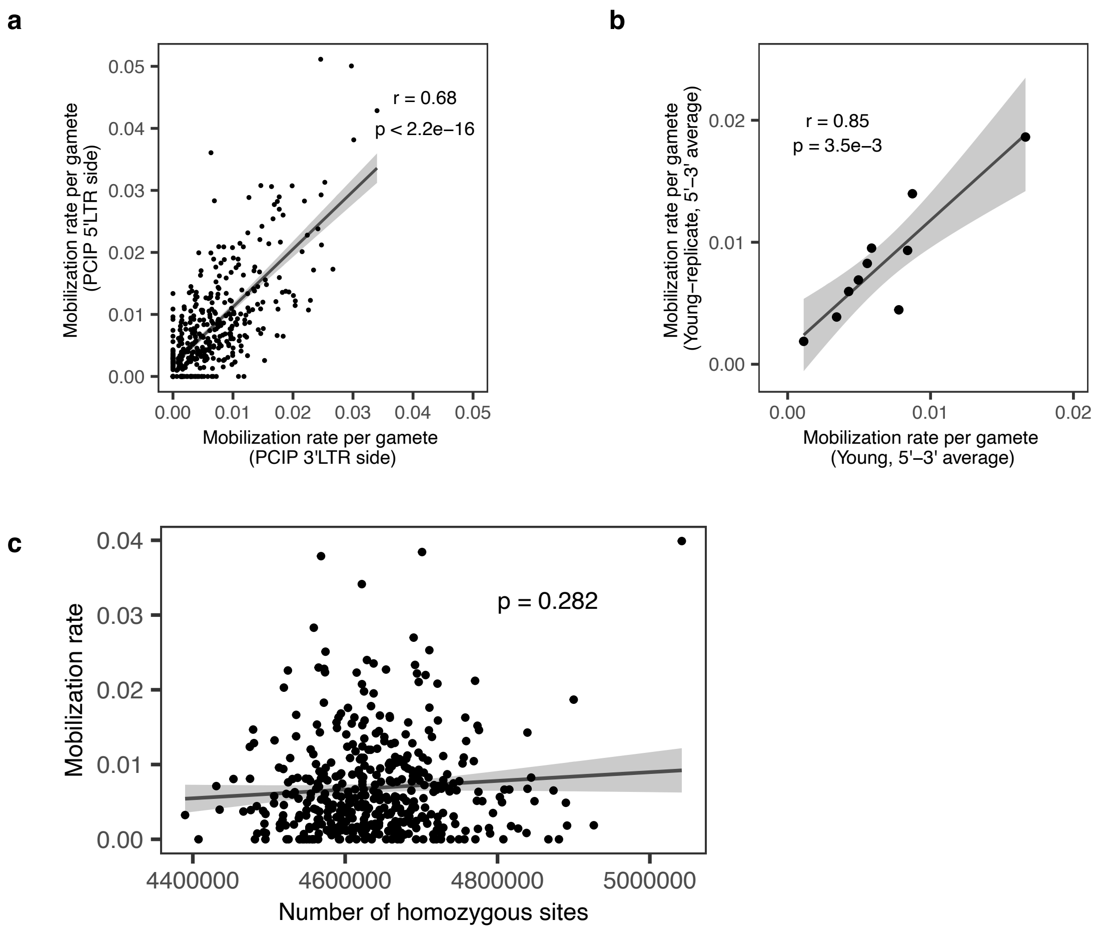

Supplementary Figure 4: Assessing the repeatability and robustness of PCIP and lack of evidence of an effect of a bull's inbreeding coefficient on the rate of ERVK[2-1-LTR] mobilization in its germline.

(a) Correlation between the 5' and 3' PCIP estimates of the mobilization rate of ERVK[2-1-LTR] in sperm DNA of 430 Belgian Blue sires. The correlation (r) of 0.68 indicates that PCIP is able to robustly measure differences in mobilization rate between samples. **(b)** Correlation between estimates of the mobilization rate of ERVK[2-1-LTR] elements in sperm samples for two biological replicates of ten young Belgian Blue bulls. Estimates correspond to the average of the 5'LTR and 3'LTR measures. Spearman's correlation (r) was 0.85. **(c)** The ERVK[2-1-LTR] mobilization rate measured in sperm of 430 Belgian Blue bulls (Y-axis) as a function of the number of homozygous sites out of 7,428,183 SNPs (X-axis) (MAF ≥ 0.1), used as proxy for their inbreeding coefficient. Source data are provided as a Source Data file.

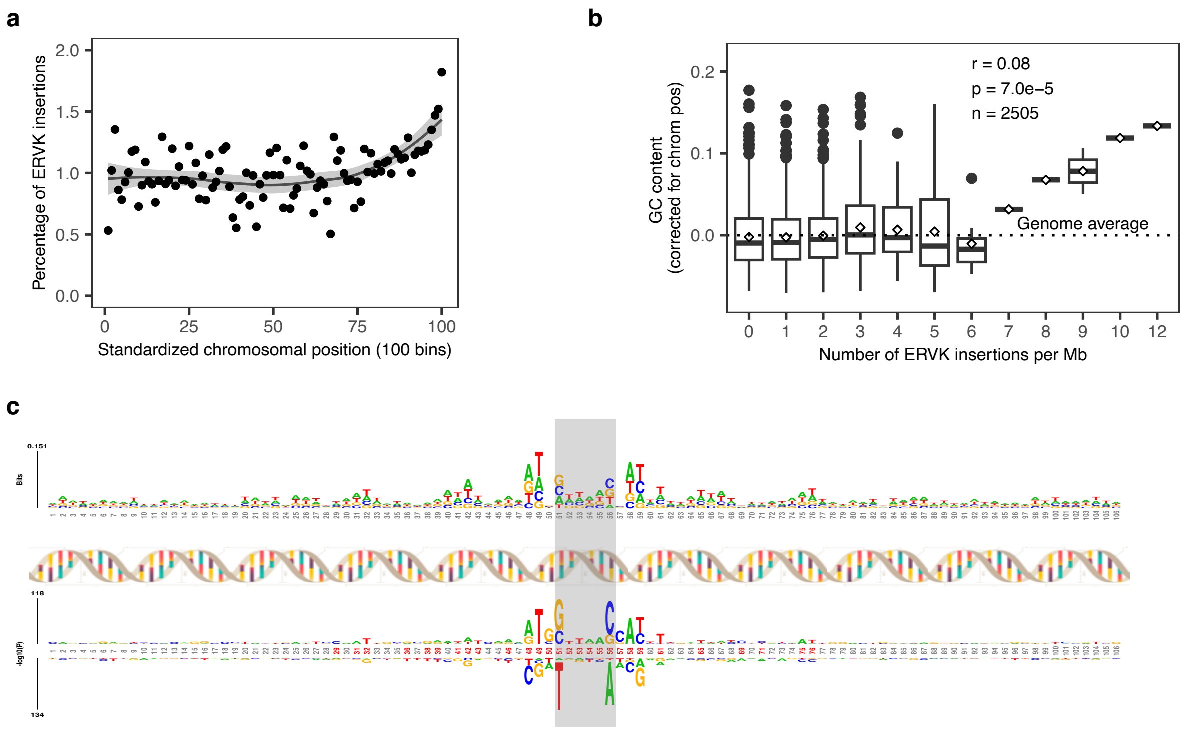

Supplementary Figure 5: Genomic landscape of ERVK[2-1-LTR] *de novo* insertions.
(a) Distribution of ERVK[2-1-LTR] insertions across standardized chromosome length. Chromosomes were split in 100 equally sized bins; the number ERVK[2-1-LTR] insertions counted for each bin, divided by the total number insertions on that chromosome, and multiplied by 100. Bin-specific percentages were then averaged across the 29 autosomes. A regression curve was fitted to the data using LOESS. ERVK[2-1-LTR] insertions appear to preferentially occur towards the telomere. Of note, all bovine autosomes are acrocentric. Thus, bin 1 is close to the centromere, and bin 100 close to the telomere. **(b)** Effect of local GC content on the rate of ERVK[2-1-LTR] insertion. The genome was subdivided in 2505 non-overlapping 1Mb bins. GC content was computed for each bin, and corrected (using a linear model) for distance from chromosome center. We then looked at the distribution of GC content for bins with 0, 1, 2, … 12 ERVK[2-1-LTR] insertions. There was a striking positive correlation between GC content and the number of insertions (r= 0.08, p=2.7x10^{-5}). A rectangle is drawn to represent the second and third quartiles with a horizontal line inside to indicate the median value. **(c)** 6-bp duplication (grey box) and 8-bp pseudo-palindromic motif at ERVK[2-1-LTR] insertion sites. Upper logo: nucleotide composition flanking 3669 ERVK[2-1-LTR] insertion sites. The height of the column corresponds to the departure from uniform nucleotide composition (measure of entropy). It shows strong signals for positions 1 (G) and 6 (C) of the duplication, as well as positions -3 (A), -2 (T), +2 (T) and +3 (A), as well as weaker up- and down-stream signals that appear to show a 10-bp (one helical turn) periodicity. Lower logo: log(1/p) value of the enrichment/depletion of the four nucleotides with respect to the average across a 100 bp window centered on the insertion. P-values were determined using z-scores computed with the mean and standard deviation of the abundance of the corresponding nucleotide in the window. The signal was strongest for positions 1 (G) and 6 (C) of the duplication, as well as positions -3 (A), -2 (T), -1 (G), +1 (C), +2 (T), +3 (A), and +5 (T). Although palindromic in appearance (5'ATGG….CCAT-3') when considering average base pair composition, there was no evidence for palindromicity when considering individual sequences as reported by Kirk et al. (2016). Logos were plotted using http://kplogo.wi.mit.edu/. Source data are provided as a Source Data file.

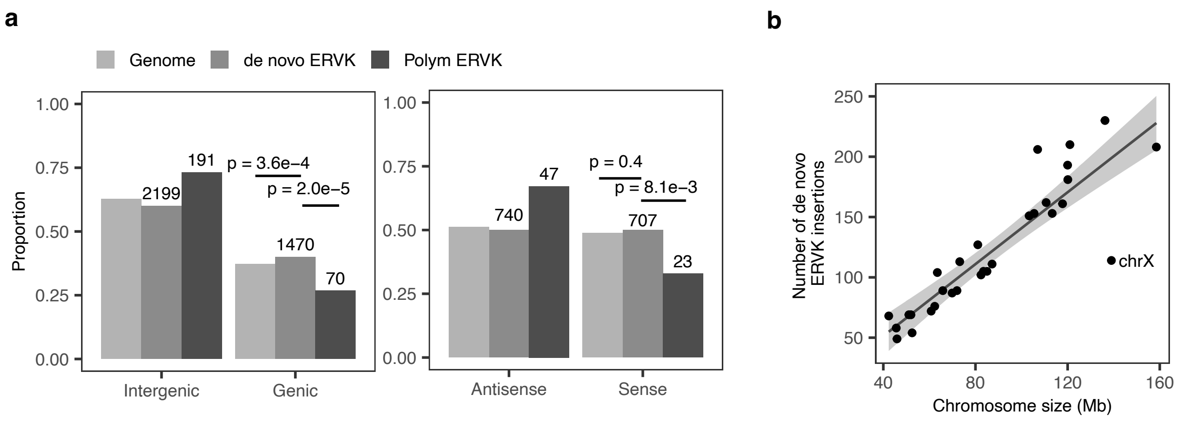

Supplementary Figure 6: Genomic features of ERVK[2-1-LTR] *de novo* insertions.
(a) Distribution of *de novo* ERVK[2-1-LTR] mobilization events with respect to genic vs intergenic and genic sense vs genic anti-sense space. We observed a modest yet significant (p = 0.0004) over-representation of genic *vs* intergenic insertions but not of anti-sense *vs* sense insertions. A number of factors may explain this observation including (i) more accessible genic than intergenic space, or (ii) overestimation of the genic space. The corresponding proportions are shown for polymorphic ERVK[2-1-LTR] elements (i.e. segregating in the Belgian Blue cattle population) for comparison. **(b)** Relationship between chromosome size and number of *de novo* ERVK[2-1-LTR] insertions. The chromosome that stands out the most is the X chromosome with nearly halve the number of insertions when compared to expectations. The most likely explanation of this observation is the hemizygosity of the X in male samples. Source data are provided as a Source Data file.

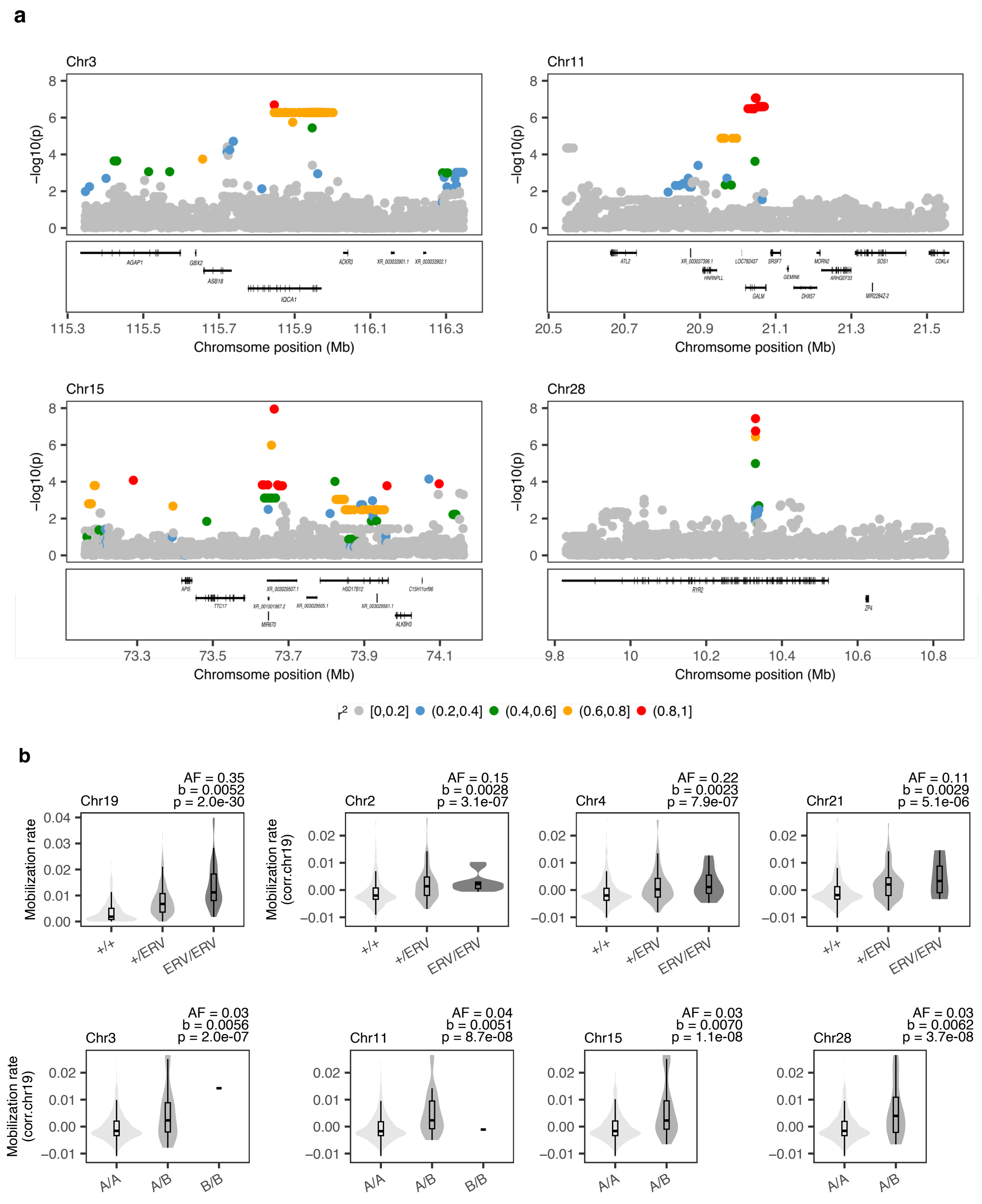

Supplementary Figure 7: Additional GWAS loci and genotypic effect for the eight significant loci.
(a) Zooms into the local association patterns of the four loci affecting ERVK[2-1-LTR] mobilization rate that do not encompass an ERV element. Variants are colored according to their LD (r^2) with the lead variant. Gene content of the corresponding window are shown below each panel. **(b)** Violin / box-plots showing the distribution of the ERVK[2-1-LTR] germline mobilization rates by marker genotype. For the four ERVK encompassing loci, bulls were sorted by ERV genotype (+/+, +/ERV, ERV/ERV). For the four other loci, bulls were

sorted based on genotype at the lead SNP. AF: allelic frequency; b(eta): effect of the allele substitution on mobilization rate (slope of the linear regression on allelic dosage); p(value): statistical significance of the association. All non-ERVK lead SNPs were imputed variants with imputation accuracy $\geq$ 0.95. A rectangle is drawn to represent the second and third quartiles with a horizontal line inside to indicate the median value. Source data are provided as a Source Data file.

a

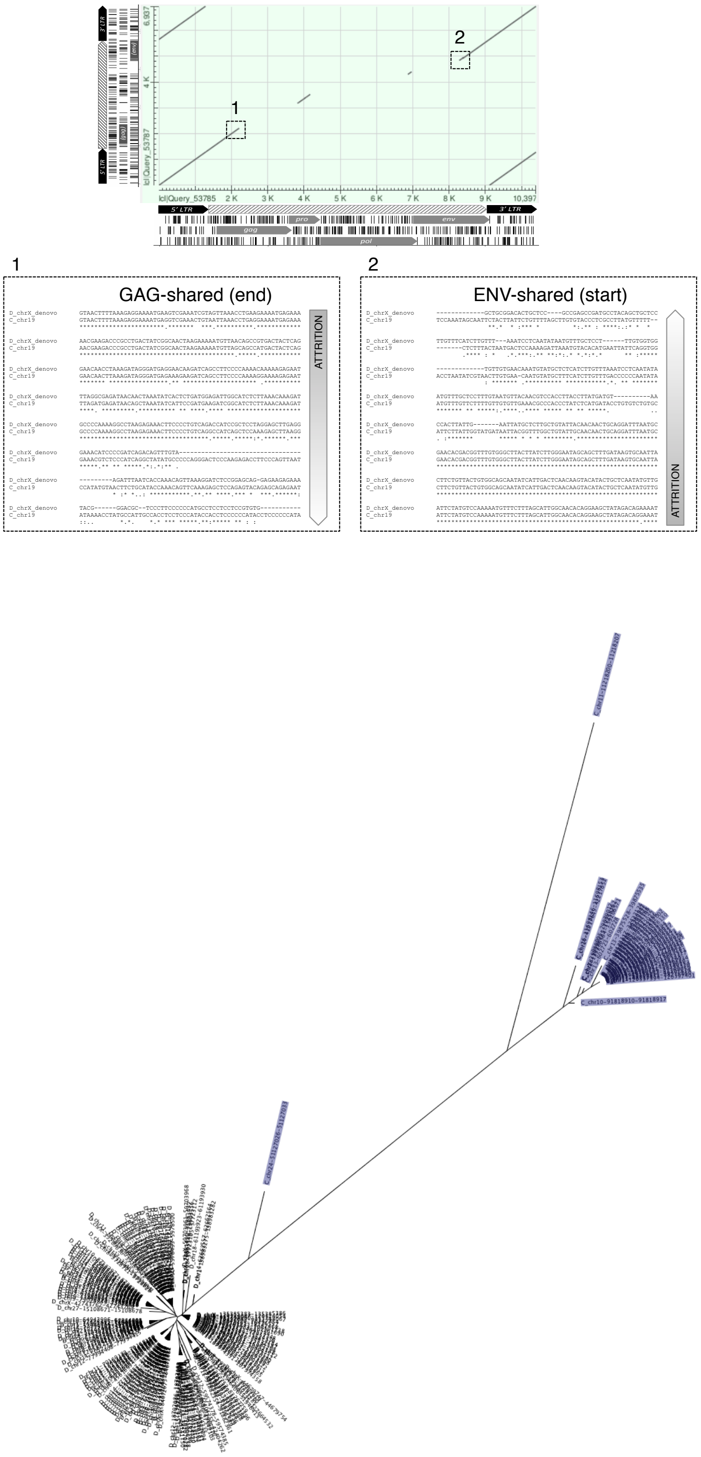

b

Supplementary Figure 8: Dot plot between sequences representing the C clade (X-axis) and D clade (Y-axis) and neighbor-joining tree obtained with the concatenated *GAG*-shared (55 base pairs) and *ENV*-shared (195 base pairs) segments.

(a) The diagonal lines correspond to nearly identical 5' and 3'LTR, shared *GAG* and *ENV* segments (extremities), as well fragments of *PRO* and *POL* (P and L in main Fig. 5). The limits of the *GAG*- (1) and *ENV*-shared (2) segments are boxed with dashed lines in the dot plot. The corresponding sequence alignments (1: *GAG*-shared; 2: *ENV*-shared) between a C

and a D ERVK[2-1-LTR] element are displayed below the dot plot. Decreasing similarity (attrition) is marked by arrows with gradients (from high to low similarity). The arrowheads mark the boundaries of the *GAG* and *ENV* shared sequences used to generate the tree shown in b. **(b)** Indeed, these segments do not show obvious "within segment" traces of recombination between C- and D-type elements so should provide the most correct relationship between C- and D-type (see hereafter for one "between segment" recombination). Insertion-deletions of more than one nucleotide were collapsed to single events. C-type elements are highlighted in blue. C- and D-type elements appear as clear distinct clades. One C-element (chr. 24) is closer to the D-clade. It corresponds to the only element that has a complete C-type *GAG*-shared sequence associated with a D-type *ENV*-shared sequence. The average pairwise difference between C- and D-type elements was 38.4 in 250 base-pairs. Assuming a *de novo* mutation rate of $1x10^{-8}$ base pairs per generation [30], this would correspond to ~15 million generations or ~50 million years, hence suggesting that the endogenization of the exogenous retrovirus precursor of the ERVK[2-1-LTR] element is a very ancient event. It is, however, possible that some of the differences between C- and D-type shared sequences were introduced upon creation of D-type elements, and/or that the mutation rate for ERV elements, influenced by the retro-transposition process, is higher than $1x10^{-8}$ base pairs per generation. Estimates of the divergence are also influenced by the somewhat arbitrary definition of the boundaries of the *GAG*-shared and *ENV*-shared segments.

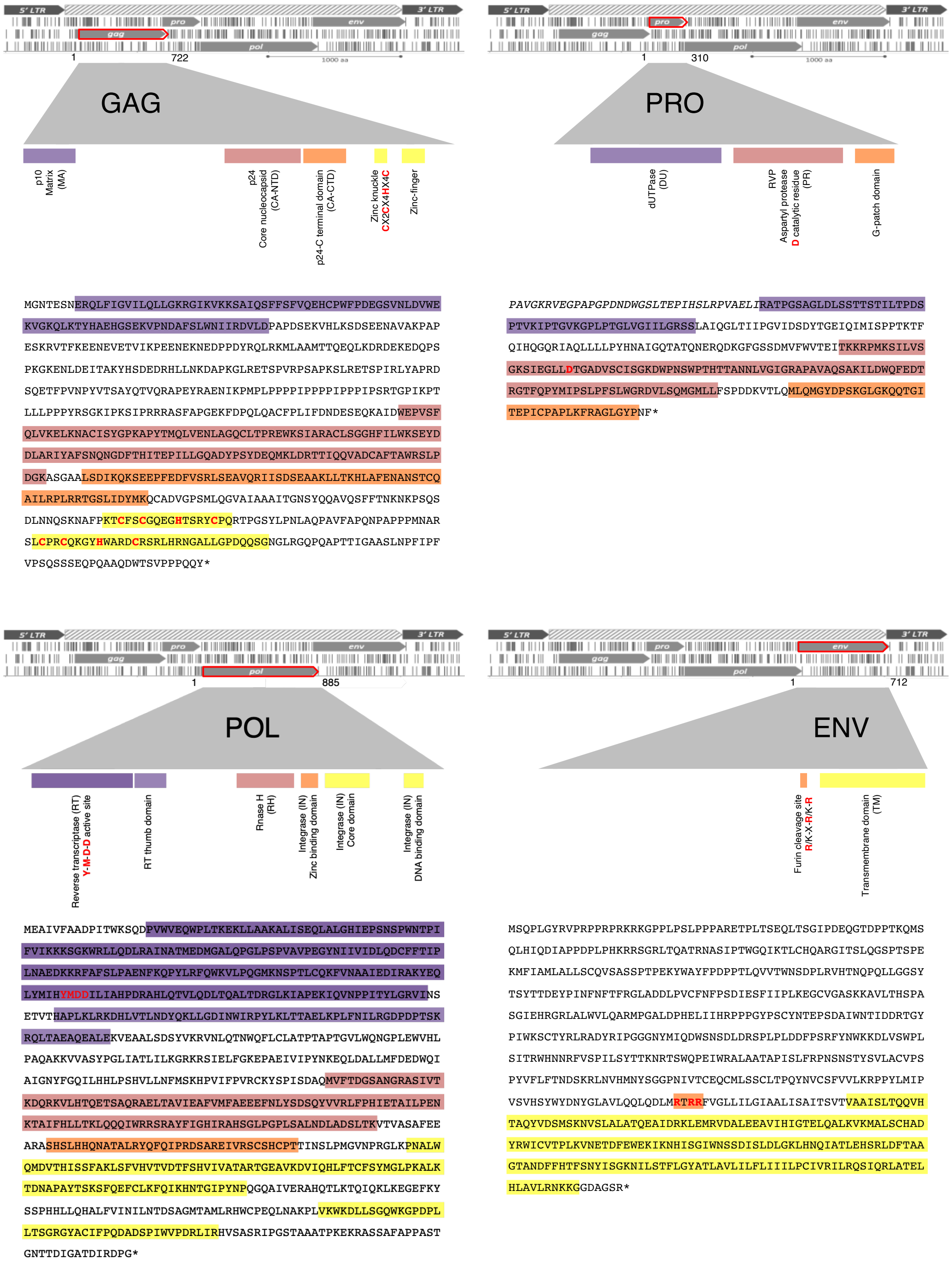

Supplementary Figure 9: Protein sequences and domain annotation of a representative element of the C-clade (chr19: 50,466,809 bp).
Schematic representation of the four ORFs' domain annotation according to specific hits and super-families obtained by blasting each ORF against 'viruses' (taxid: 10239) non-redundant

protein sequences. Number of amino-acids is given above each ORF (GAG, 722; PRO, 310; POL, 885; ENV, 712). The corresponding amino acid sequence is highlighted with the same color code below each domain scheme. Amino acids in italics at the N-terminal part of the PRO protein correspond to putative translation start sites. See also Supplementary Table 1 for a more detailed description of each protein domain.

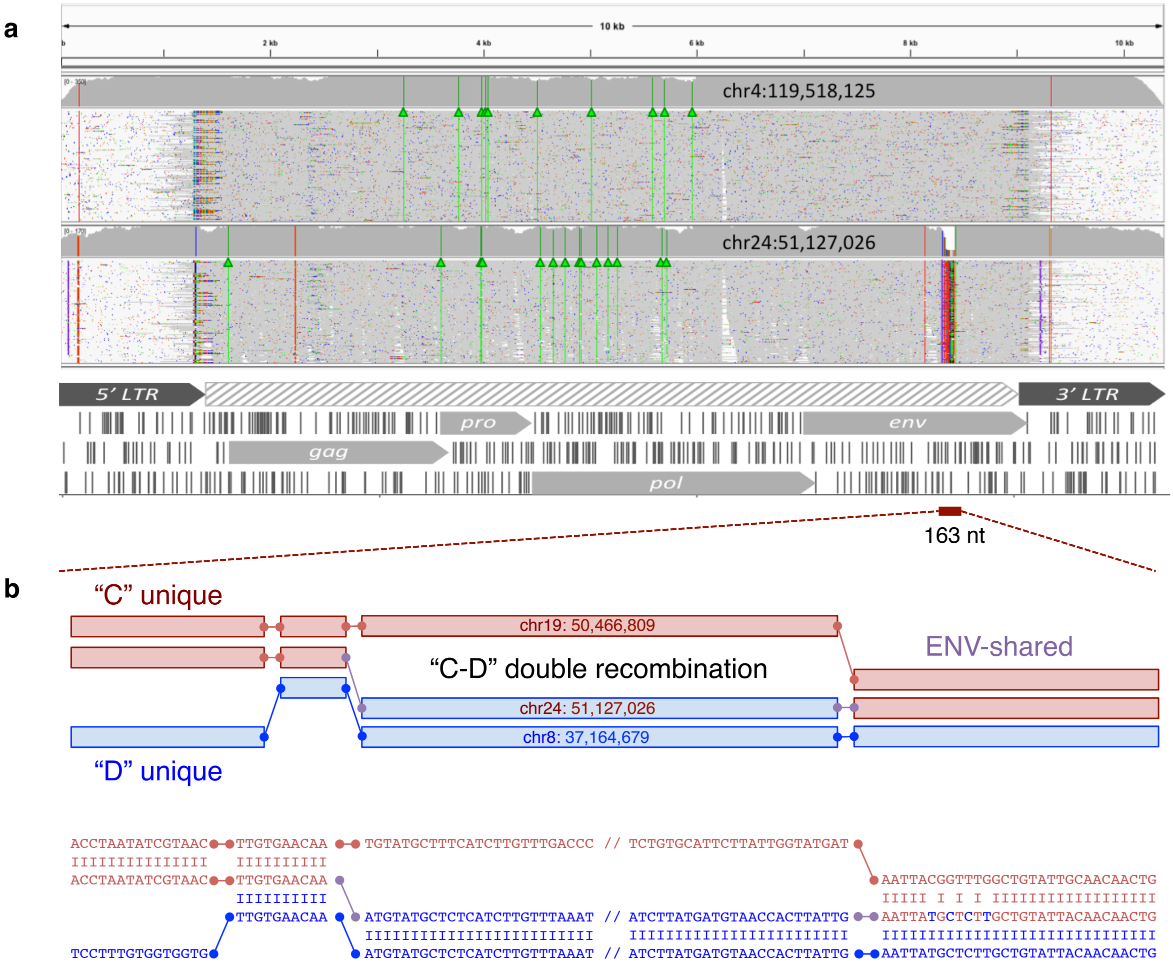

Supplementary Figure 10: Examples of APOBEC3 mutational signature, recombination events and attrition at the boundaries.
(a) Illustration of APOBEC3 induced G-to-A transitions on the plus-strand of the ERVK[2-1-LTR] genetic code: IGV screen capture of genomic sequence reads obtained from PCR amplicons of two clade 'C' ERVK[2-1-LTR] elements (chr4:119,518,125: top panel and chr24:51,127,026: bottom panel) mapped on the ~10Kb (5' to 3') reference genomic sequence of a representative clade 'C' element (chr19: 50,466,809; top GWAS signal). Green vertical bars correspond to G-to-A transition and each one is highlighted by a green triangle (n = 11, chr4; n = 16, chr24). Other polymorphisms are shown with vertical color bars. Size and location of a double recombination are positioned by a red horizontal bar (163nt). **(b)** Schematic representation of a 163nt double recombination event between a clade 'C' (chr19: 50,466,809; top connected red rectangles) and a clade 'D' (chr8: 37,164, 679; bottom connected blue rectangles) ERVK[2-1-LTR] elements resulting in a recombinant 'C' element (chr24:51,127,026; middle connected red-blue-red rectangles). Their corresponding genomic sequence is given below with the same order and color code.

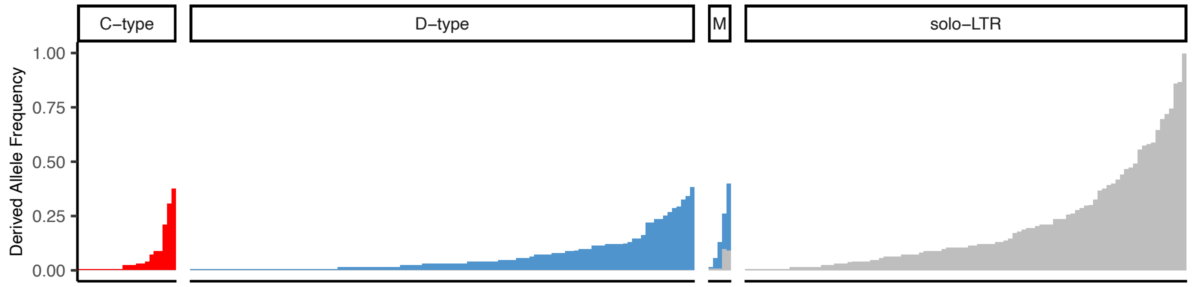

Supplementary Figure 11: Distribution of allelic frequencies of ERVK[2-1-LTR] segregating in Belgian Blue cattle.
ERVK[2-1-LTR] elements are sorted by “morph”: C-type, D-type, solo-LTR. Within morph, the ERVK[2-1-LTR] elements are ranked by their frequency in the Belgian Blue population. At five loci, solo-LTR, D-type and wild-type (+, grey) alleles coexist at the shown frequencies (M(ultiple) morph). Source data are provided as a Source Data file.

a

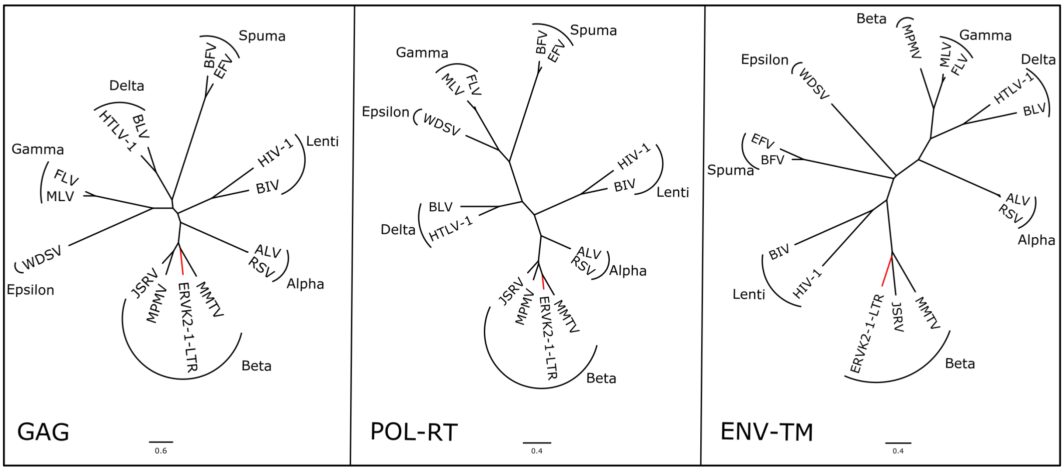

b

MMTV	M	G	V	S	G	S	K	G	Q	K	L	F	V	S	V	L	Q	R	L	L	-	-	0.9753
MPMV	M	G	Q	E	L	S	Q	-	H	E	R	Y	V	E	Q	L	K	Q	A	L	K	-	0.9839
JSRV	M	G	Q	T	H	S	-	-	R	Q	L	F	V	H	M	L	S	V	M	L	K	H	0.9781
ERVK2-1-LTR	M	G	N	T	E	S	N	E	R	Q	L	F	I	G	V	I	L	Q	L	L	-	-	0.9838

c

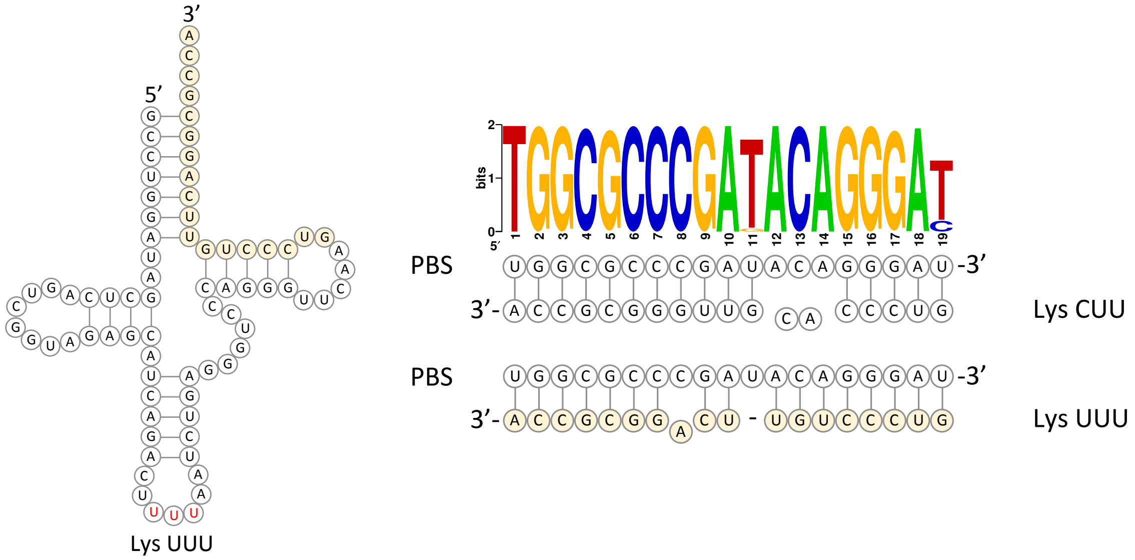

Supplementary Figure 12: Phylogenetic relationship with exogenous retroviruses and functional sequence features of ERVK[2-1-LTR].

(a) Unrooted phylogenetics trees of GAG, POL (reverse transcriptase domain) and ENV (transmembrane domain) sequences of ERVK[2-1-LTR] and representative Alpha, Beta, Gamma, Delta, Epsilon, Lenti and Spuma exogenous retroviruses. ALV: Avian leukosis virus; RSV: Rous sarcoma virus; MMTV: Mouse mammary tumor virus; MPMV: Mason-Pfizer monkey virus; JSRV: Jaagsiekte sheep retrovirus; HTLV-1: Human T-lymphotropic virus 1; BLV: Bovine leukemia virus; WDSV: Walleye dermal sarcoma virus; MLV: Moloney murine leukemia virus; FLV: Feline leukemia virus; HIV-1: Human immunodeficiency virus 1;BIV: Bovine immunodeficiency virus; BFV: Bovine foamy virus; EFV: Equine foamy virus. **(b)** Sequence of the N-terminal domain of the representative beta retroviruses and ERVK[2-1-LTR] GAG protein disclosing a consensus sequence required for myristoylation ([M]GXXXS/T) with the first M corresponding to the GAG initiation codon and a domain

rich in positively charged basic residues (basic domain in red) interacting with the membrane phospholipids. The score for myristoylation was predicted by Myristoylator (https://web.expasy.org/cgi-bin/myristoylator). **(c)** Consensus primer binding site (PBS) shared by C and D-type elements aligned to the 3' end of the bovine CTT and TTT lysine tRNA genes.

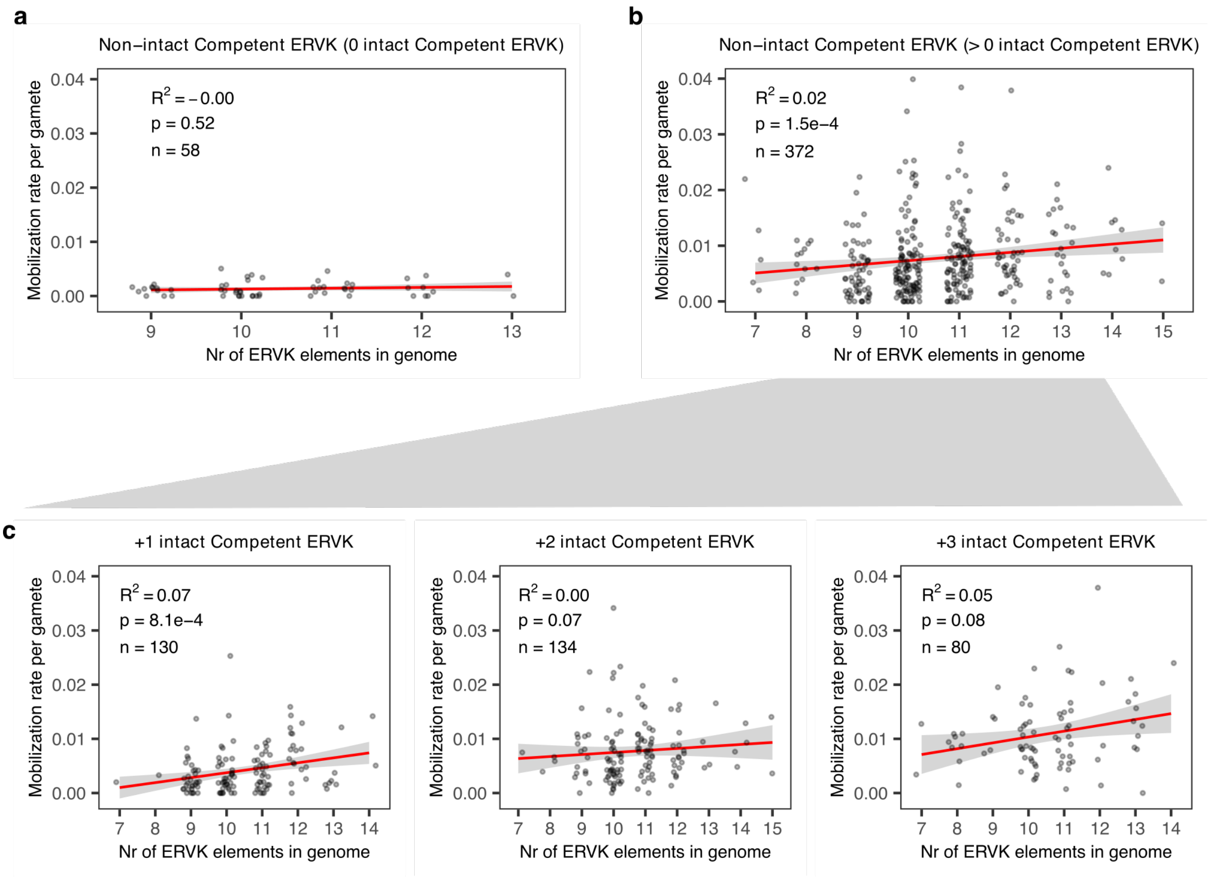

Supplementary Figure 13: Epistatic interaction between ERVK[2-1-LTR] elements with coding variants in the *GAG*, *PRO*, *POL* or *ENV* gene, and ERVK[2-1-LTR] elements without.

(a) In the absence of any intact Competent ERVK[2-1-LTR] element, the dosage (0, 1 or 2) of ERVK[2-1-LTR] elements with coding variants has no effect (p = 0.693) on the *de novo* mobilization rate. **(b)** With one or more intact Competent ERVK[2-1-LTR] elements in the genome, the dosage (0, 1 or 2) of ERVK[2-1-LTR] elements with coding variants has a significant effect (p = 7.0e-04) on the *de novo* mobilization rate. The probability to have a regression slope as low as the one observed without intact Competent ERVK[2-1-LTR] elements, assuming that the effect of dosage is in fact the same as in the presence of intact Competent ERVK[2-1-LTR] elements, i.e. the probability that it is a false negative was shown to be 0.11. **(c)** Effects of the number of non-intact ERVK[2-1-LTR] elements in the presence of 1, 2 or 3 intact ERVK[2-1-LTR] elements are shown separately. Source data are provided as a Source Data file.

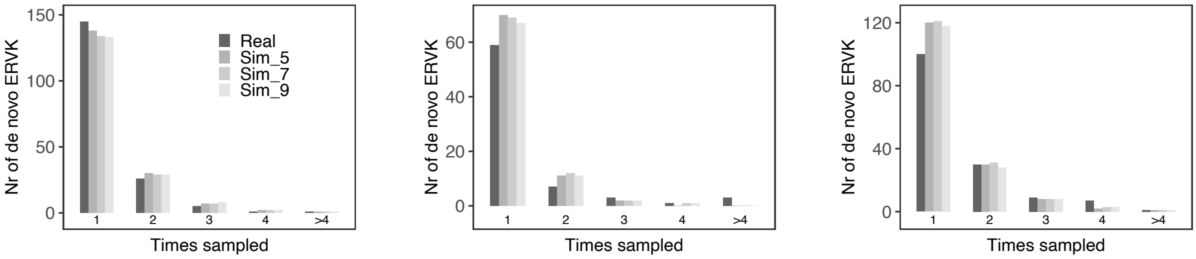

Supplementary Figure 14: Comparison between the real (dark grey) and simulated (lighter greys) frequency distribution of the resampling rate of ERVK[2-1-LTR] *de novo* mobilization events in BB bulls BE157971524, BE187351114, BE63811423.
The simulations assumed that mobilization occurred in a developmental window centered on cell generation 14 of spermatogenesis of 21 (yielding 1,048,576 spermatogonial stem cells). Windows spanning 5, 7 or 9 cell generations, with mobilization rates distributed as shown in Fig. 8b, matched the real data (3 bulls combined) equally well. The simulations further matched the real data with regards to bull-specific *de novo* insertion frequency (average number of *de novo* insertions per sperm cell) and number of explored haploid genomes (see Methods). Source data are provided as a Source Data file.

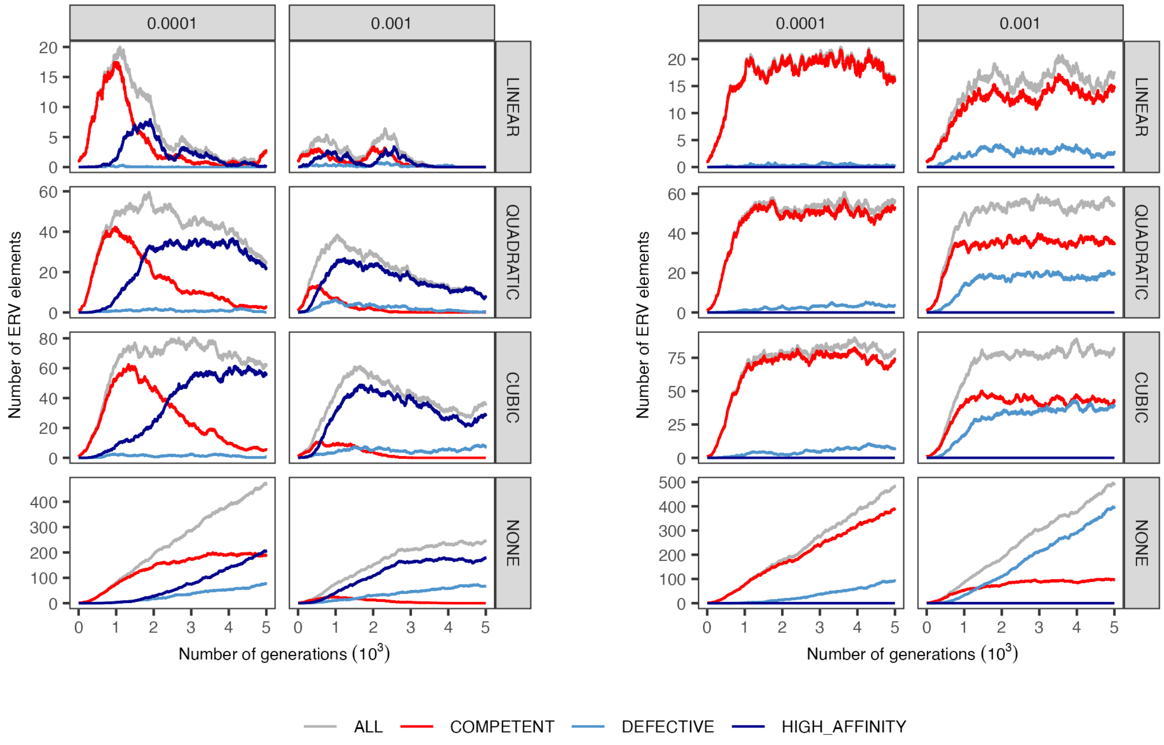

Supplementary Figure 15: Unselected examples of the evolution of the number of ERV elements (per genome) in a panmictic population of 1,000 animals over a course of 5,000 generations.

In the left panel, mutation may cause competent elements to become defective and either increase or decrease the affinity for the mobilization machinery (provided in *trans* by competent elements). In the right panel, mutation can only cause competent elements to become defective and lose their affinity for the mobilization machinery. The mutation rate was set at 0.0001 and 0.001 per element per generation. Purifying selection was set from severe ("linear") to none ("none") with two intermediate intensities ("quadratic" and "cubic"). Source data are provided as a Source Data file.

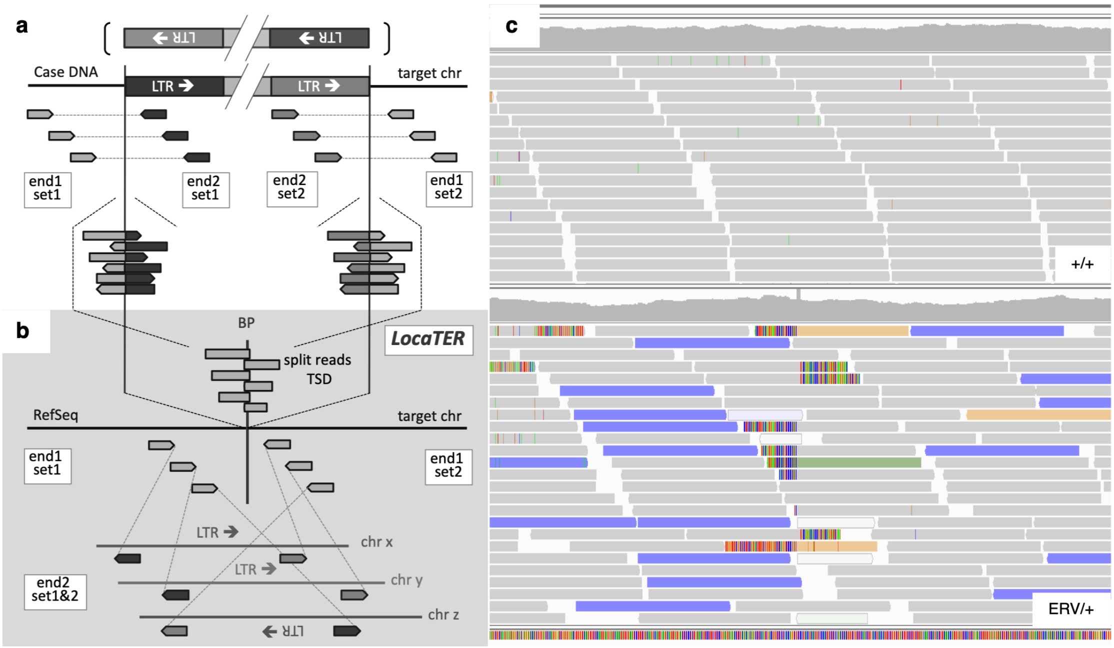

Supplementary Figure 16: Schematic representation of the features exploited by *LocaTER* to identify polymorphic ERV element absent from the bovine reference sequence.

(a) Representation of the features of an ERV insertion when the WGS paired reads are (hypothetically) mapped on the case DNA sequence. **(b)** Representation of the features of an ERV when the WGS paired reads are mapped on the reference sequence. **(c)** IGV screen capture of homozygous wide type (upper) and one heterozygous ERV insertion (bottom) detected by *LocaTER*. *LocaTER* exploits three distinctive features of ERV element insertion: (i) paired sets of discordant paired-ends (with respect to ARS-UCD1.2 genome assembly) mapping, respectively, to the sense strand upstream the insertion site (end 1, set 1) and one end of the ERV LTR (end 2, set 1), and to the antisense strand downstream of the insertion of the insertion site (end 2, set 2) and the other end of the LTR (end 1, set 2), (ii) the presence of split sense and antisense reads consistently bridging the insertion site and the ERV LTR, (iii) the presence of the signature target site duplication (TSD). Breakpoint (BP) at the insertion site is represented by a vertical bar.

Supplementary Table 1: Detailed domain annotation of a representative element of the C-clade (chr19: 50,466,809 bp).
Specific hits and super-families were obtained by blasting each ORF (GAG, PRO, POL, ENV) against 'viruses' (taxid: 10239) non-redundant protein sequences.

GAG_722	Name	Accession	Description	Interval	E-value
	Gag_p24	pfam00607	gag gene protein p24 (core nucleocapsid protein, CA) ;p24 forms inner protein layer of the nucleocapsid	348-539	5.06E-50
	Gag_p10	pfam02337	Retroviral GAG p10 protein; This family consists of various retroviral GAG (core) polyproteins and encompasses the p10 region producing the p10 protein upon proteolytic cleavage of GAG by retroviral protease. The p10 or matrix protein (MA) is associated with the virus envelope glycoproteins in most mammalian retroviruses and may be involved in virus particle assembly, transport and budding.	10-92	3.87E-37
	zf-CCHC_5	pfam14787	GAG-polyprotein viral zinc-finger.	640-672	5.00E-09
	PTZ00368	PTZ00368	universal minicircle sequence binding protein (UMSBP).	592-654	4.63E-05
	ZnF_C2HC	smart00343	zinc finger;	593-609	4.12E-03

PRO_310	Name	Accession	Description	Interval	E-value
	RVP	pfam00077	Retroviral aspartyl protease; Single domain aspartyl proteases (PR) from retroviruses, retrotransposons, and badnaviruses (plant dsDNA viruses). These proteases are generally part of a larger polyprotein; usually pol, more rarely gag.	163-261	3.91E-27
	HIV_retropepsin_like	cd05482	Retropepsins, pepsin-like aspartate proteases (PR); This is a subfamily of retropepsins. The family includes pepsin-like aspartate proteases from retroviruses, retrotransposons and retroelements. While fungal and mammalian pepsins are bilobal proteins with structurally related N- and C-termini, retropepsins are half as long as their fungal and mammalian counterparts. The monomers are structurally related to one lobe of the pepsin molecule and retropepsins function as homodimers. The active site aspartate (D) occurs within a motif (Asp-Thr/Ser-Gly), as it does in pepsin. Retroviral aspartyl protease is synthesized as part of the POL polyprotein that contains an aspartyl protease, a reverse transcriptase, RNase H, and an integrase. The POL polyprotein undergoes specific enzymatic cleavage to yield the mature proteins. In aspartate peptidases, Asp residues are ligands of an activated water molecule in all examples where catalytic residues have been identified.	170-255	3.15E-26
	G-patch	pfam01585	G-patch domain; This domain is found in a number of RNA binding proteins, and is also found in proteins that contain RNA binding domains. This suggests that this domain may have an RNA binding function. This domain has seven highly conserved glycines.	272-308	1.34E-06
	dUTPase	pfam00692	dUTPase (DU); dUTPase hydrolyzes dUTP to dUMP and pyrophosphate.	36-153	2.28E-35

POL_886	Name	Accession	Description	Interval	E-value
	RT_Rtv	cd01645	RT_Rtv: Reverse transcriptases (RTs) from retroviruses (Rtvs). RTs catalyze the conversion of single-stranded RNA into double-stranded viral DNA for integration into host chromosomes. Proteins in this subfamily contain long terminal repeats (LTRs) and are multifunctional enzymes with RNA-directed DNA polymerase, DNA directed DNA polymerase, and ribonuclease hybrid (RNase H) activities. The viral RNA genome enters the cytoplasm as part of a nucleoprotein complex, and the process of reverse transcription generates in the cytoplasm forming a linear DNA duplex via an intricate series of steps.	18-230	8.70E-119
	RVT_thumb	pfam06817	Reverse transcriptase thumb domain; This domain is known as the thumb domain. It is composed of a four helix bundle.	237-302	5.43E-36
	RNase_HI_RT_Bel	cd09273	Bel/Pao family of RNase HI in long-term repeat retroelements; Ribonuclease H (RNase H) enzymes are divided into two major families, Type 1 and Type 2, based on amino acid sequence similarities and biochemical properties. RNase H is an endonuclease that cleaves the RNA strand of an RNA/DNA hybrid in a sequence non-specific manner in the presence of divalent cations. RNase H is widely present in various organisms, including bacteria, archaea and eukaryote. RNase HI has also been observed as adjunct domains to the reverse transcriptase gene in retroviruses, in long-term repeat (LTR)-bearing retrotransposons and non-LTR retrotransposons. RNase HI in LTR retrotransposons perform degradation of the original RNA template, generation of a polypurine tract (the primer for plus-strand DNA synthesis), and final removal of RNA primers from newly synthesized minus and plus strands. The catalytic residues for RNase H enzymatic activity, three aspartatic acids and one glutamic acid residue (DEDD), are unvaried across all RNase H domains. Phylogenetic patterns of RNase HI of LTR retroelements is classified into five major families, Ty3/Gypsy, Ty1/Copia, Bel/Pao, DIRS1 and the vertebrate retroviruses. Bel/Pao family has been described only in metazoan genomes. RNase H inhibitors have been explored as an anti-HIV drug target because RNase H inactivation inhibits reverse transcription.	449-571	1.63E-26
	rve	pfam00665	Integrase core domain (IN);Integrase mediates integration of a DNA copy of the viral genome into the host chromosome. Integrase is composed of three domains. The amino-terminal domain is a zinc binding domain pfam02022. This domain is the central catalytic domain. The carboxyl terminal domain that is a non-specific DNA binding domain pfam00552. The catalytic domain acts as an endonuclease when two nucleotides are removed from the 3' ends of the blunt-ended viral DNA made by reverse transcription. This domain also catalyzes the DNA strand transfer reaction of the 3' ends of the viral DNA to the 5' ends of the integration site	633-727	7.17E-24
	IN_DBD_C	pfam00552	Integrase DNA binding domain (IN); Integrase mediates integration of a DNA copy of the viral genome into the host chromosome. Integrase is composed of three domains. The amino-terminal domain is a zinc binding domain. The central domain is the catalytic domain pfam00665. This domain is the carboxyl terminal domain that is a non-specific DNA binding domain.	796-839	5.02E-18

ENV_713	Name	Accession	Description	Interval	E-value
	GP41	pfam00517	Retroviral envelope protein; This family includes envelope protein from a variety of retroviruses. It includes the GP41 subunit of the envelope protein complex from human and simian immunodeficiency viruses (HIV and SIV) which mediate membrane fusion during viral entry. The family also includes bovine immunodeficiency virus, feline immunodeficiency virus and Equine infectious anaemia (EIAV). The family also includes the Gp36 protein from mouse mammary tumor virus (MMTV) and human endogenous retroviruses (HERVs).	512-706	1.34E-35

Supplemental Method 1: *LocaTER* for the detection of polymorphic and *de novo* repeat insertions.

The *LocaTER* pipeline is designed to identify candidate polymorphic ERV insertions from next generation WGS data (Supplementary Fig. 16). It requires a database of locations for annotated ERV in the reference genome, Ensembl and RefSeq transcript databases, individual sorted Illumina paired end BAM files aligned with BWA MEM and a pedigree file for the population. *LocaTER* proceeds to scan individual BAM files analyzing each read to identify the signatures of an ERV insertion. For every read in the genome it checks to determine if it is cleanly aligned (mapping quality 20-60), if the read is properly paired (SAM flag is Properly Paired), is not aligned to a known ERV and that the mate is aligned to a known ERV of a specific class. When a read is detected that matches these criteria a 1.5kb window (3x the library insert size) is created starting from that read. The software then proceeds to record key information about reads within this window. It records the total number of reads within the window, identifies all improperly paired, soft- and hard-clipped reads recording their orientation with regards to the reference genome (5' or 3'). For hard- and soft-clipped reads, it analyses the read recording the exact genomic position the read clips. For the improperly paired mates it records their orientation with regards to the reference genome, the orientation with regards to the ERV they are aligned to, and the ERV family. Once the end of the window is reached the number of observed 5' and 3' improperly paired reads and the total number of clipped reads are tested to determine if they are significantly different from the genome average for a 1.5kb window. If the observations are significantly different from the genome average the window is reported along with the recorded statistics for the window. Once all individuals are analyzed the data is combined and the data for each window is merged, if windows overlap by 500bp and share at least one clipping site they are merged. The merged windows are tested for significance and checked to ensure that the total number of improperly paired reads in both the 5' and 3' is compatible with the insertion of a single ERV (either heterozygous or homozygous) in that window, considering the number of individuals who shared the site. The difference between the two most common split read locations is calculated to identify the likely insertion site and the size of the associated target site duplication, sites with a difference greater than 20bp are discarded. A 1.5kb window is recalculated from the likely insertion site and all individual BAM files are reanalyzed for the new window collecting the data as described above. In addition, the number of reads that completely bridge the insertion site are determined and used to estimate if the site is heterozygous (1 or more read completely bridging the 5' and 3' insertion sites) or

homozygous (no reads bridging the 5' and 3' insertion sites) in the individual. The number of 5' improperly paired reads, and 3' improperly paired reads are then tested against the genome average for significance and the site retained if either are significantly different. Each site is annotated with any gene it overlaps, and the ERV class with the most mates aligned to it is selected as the likely class of the new ERV insertion. The pedigree for the population is then analyzed to identify trios and each site is checked for any violations of Mendelian inheritance (absent in both parents but present in the proband). Each site is then reported with the associated statistics and list of identified carriers, along with their likely genotype.
