# Supplementary material for: GWAS reveals determinants of mobilization rate and dynamics of an active endogenous retrovirus of cattle
Source: Nat Commun. 2024 Mar 9;15:2154. doi: 10.1038/s41467-024-46434-1 (PMC10924933; doi:10.1038/s41467-024-46434-1)
Supplement: Supplementary file 4 — Description of Additional Supplementary Files [file 41467_2024_46434_MOESM4_ESM.pdf]

Description of Additional Supplementary Files

Supplementary Data 1
Description: 1,222 polymorphic ERV elements detected by *LocaTER* in the whole genome sequences of the Damona pedigree with corresponding genotypes for all Damona animals.

Supplementary Data 2
Description: List of genes involved in control of ERV mobilization and examined in bull transmitting three *de novo* ERVK[2-1-LTR] insertions [17].

Supplementary Data 3
Description: Estimated *de novo* mobilization rate of ERVK[2-1-LTR] elements in sperm samples collected at >= 7 years interval for 10 Belgian Blue bulls. Technical replicates were realized for the samples collected at young age ("young-replicate"). Beta_5 and Beta_3 correspond to the number of explored genome estimated as described in Methods for the 5'LTR and 3'LTR reaction, respectively. dnTC_5 and dnTC_3 correspond to the number of *de novo* mobilization events detected with the 5'LTR and 3'LTR reaction, respectively. dnTR_5 and dnTR_3 are estimated of the *de novo* mobilization rate per haploid genome (dnTC/Beta) for the 5'LTR and 3'LTR reaction, respectively. dnTR is the average of dnTR_5 and dnTR_3.

Supplementary Data 4
Description: As in Supplementary Data 2 for a single sperm sample collected for 430 Belgian Blue bulls.

Supplementary Data 5
Description: The ERVK[2-1-LTR] mobilization rate is stable over time, and varies between animals. List of *de novo* mobilization events detected in sperm samples of bulls collected at young (Y) and older (O) age, with a technical replicate for the young samples (YR). We provide the number of distinct shearing sites (SS) that are detected for each *de novo* event as well as the number of reads supporting that insertion (reads). *De novo* insertions which are captured at both young and old age ("recapture") are highlighted.

Supplementary Data 6
Description: List of 3,669 *de novo* ERVK[2-1-LTR] insertions detected in sperm samples from 430 Belgian Blue bulls.

Supplementary Data 7
Description: Genotypes of 430 Belgian Blue bulls for 306 polymorphic ERVK[2-1-LTR] elements.

Supplementary Data 8
Description: 309 ERVK[2-1-LTR] elements endogenized in the Belgian Blue Cattle genome of which 306 were found to be polymorphic.

Supplementary Data 9
Description: Intersection of polymorphic ERVK[2-1-LTR] loci detected by *LocaTER* and PCIP for 40 whole genome sequenced bulls. Five loci (PCR validated), highlighted in yellow, are characterized by cosegregating solo-LTR and full-length ERV alleles cosegargating. Eighty four of 100 putative solo-LTR only loci, that were only detected by *LocaTER*, were confirmed by PCR.

Supplementary Data 10
Description: Annotation and features of 53 *C*-type ERVK[2-1-LTR] elements. DAF: derived allele frequency in BB cattle. PBS: Primer binding site. LS: Leader sequences, come into two forms (1: ~250bp and ~2: 420 bp). GAG, PRO, POL ENV: coding variants in the four respective open reading frames.

Supplementary Data 11
Description: PCIP Tag information for 309 PCIP detectable ERTVK[2-1-LTR] elements in 430 Belgian Blue bulls.

Supplementary Data 12
Description: Categories of four ERV-Tags for both endogenous and *de novo* ERVK[2-1-LTR] detected in three studied bulls.

Supplementary Data 13
Description: Results of the simulations of *de novo* mobilization occurring in windows of 1 to 9 (sim_1, sim_3, sim_5, sim_7, sim_09) consecutive cell generations, centered on "focal" generation 1 to 21 of 21 (yielding 2^20 or cells 1,048,576 spermatogonial stem cells. The first column in each block ("REAL DATA") shows the number of *de novo* insertions that were (re)captured 1, 2, 3, 4 or >4 times, and this for each one of the three studied bulls (BE157971524, BE187351114, BE63811423). The next 21 columns show the number of *de novo* events that were recaptured by simulations using "cell generation windows" of the size corresponding to the block (1, 3, 5, 7 or 9), and centered on the cell generation marked by "Mx".

Supplementary Data 14
Description: Detection of C-type elements in Bovinae by blasting (blastn) the full length C-type 'chromosome 19' proviral sequence against whole genome sequences available at: https://www.ncbi.nlm.nih.gov/datasets/taxonomy/tree/?taxon=9895.

Supplementary Data 15
Description: Oligos used in this study.
